# Trends of Exposure to Acrylamide as Measured by Urinary Biomarkers Levels within the HBM4EU Biomonitoring Aligned Studies (2000–2021)

**DOI:** 10.3390/toxics10080443

**Published:** 2022-08-02

**Authors:** Michael Poteser, Federica Laguzzi, Thomas Schettgen, Nina Vogel, Till Weber, Aline Murawski, Phillipp Schmidt, Maria Rüther, Marike Kolossa-Gehring, Sónia Namorado, An Van Nieuwenhuyse, Brice Appenzeller, Edda Dufthaksdóttir, Kristín Olafsdóttir, Line Småstuen Haug, Cathrine Thomsen, Fabio Barbone, Valentina Rosolen, Loïc Rambaud, Margaux Riou, Thomas Göen, Stefanie Nübler, Moritz Schäfer, Karin H. A. Zarrabi, Liese Gilles, Laura Rodriguez Martin, Greet Schoeters, Ovnair Sepai, Eva Govarts, Hanns Moshammer

**Affiliations:** 1 Department of Environmental Health, Center for Public Health, Medical University of Vienna, Kinderspitalgasse 15, 1090 Vienna, Austria; hanns.moshammer@meduniwien.ac.at; 2 Unit of Cardiovascular and Nutritional Epidemiology, Institute of Environmental Medicine, Karolinska-Institutet, Nobels väg 13, Box 210, 17177 Stockholm, Sweden; federica.laguzzi@ki.se; 3 Institute for Occupational, Social and Environmental Medicine, Medical Faculty, RWTH Aachen University, Pauwelsstrasse 30, 52074 Aachen, Germany; tschettgen@ukaachen.de; 4 German Environment Agency (UBA), 14193 Berlin, Germany; nina.vogel@uba.de (N.V.); till.weber@uba.de (T.W.); aline.murawski@uba.de (A.M.); phillipp.schmidt@uba.de (P.S.); maria.ruether@uba.de (M.R.); marike.kolossa@uba.de (M.K.-G.); 5 Department of Epidemiology, National Institute of Health Dr. Ricardo Jorge, 1649-016 Lisbon, Portugal; sonia.namorado@insa.min-saude.pt; 6 Laboratoire National de Santé (LNS), L-3555 Dudelange, Luxembourg; an.vannieuwenhuyse@lns.etat.lu; 7 Department of Precision Health, Luxembourg Institute of Health (LIH), L-4354 Esch-sur-Alzette, Luxembourg; brice.appenzeller@lih.lu; 8 Faculty of Food Science and Nutrition, School of Health Sciences, University of Iceland, 102 Reykjavik, Iceland; eddaduf@gmail.com; 9 Department of Pharmacology and Toxicology, University of Iceland, 120 Reykjavik, Iceland; stinaola@hi.is; 10 Norwegian Institute of Public Health, Lovisenberggata 8, 0456 Oslo, Norway; linesmastuen.haug@fhi.no (L.S.H.); cathrine.thomsen@fhi.no (C.T.); 11 Department of Medical Area, DAME, University of Udine, 33100 Udine, Italy; fabio.barbone@uniud.it; 12 Institute for Maternal and Child Health-IRCCS “Burlo Garofolo”, 34137 Trieste, Italy; valentina.rosolen@burlo.trieste.it; 13 Santé Publique France, French Public Health Agency (ANSP), 94415 Saint-Maurice, France; loic.rambaud@santepubliquefrance.fr (L.R.); margaux.riou@santepubliquefrance.fr (M.R.); 14 Institute and Outpatient Clinic of Occupational, Social and Environmental Medicine, Friedrich-Alexander Universität Erlangen-Nürnberg, Henkestraße 9-11, 91054 Erlangen, Germany; thomas.goeen@fau.de (T.G.); stefanie.nuebler@fau.de (S.N.); moritz.schaefer@fau.de (M.S.); karin.ha.zarrabi@fau.de (K.H.A.Z.); 15 VITO Health, Flemish Institute for Technological Research (VITO), 2400 Mol, Belgium; liese.gilles@vito.be (L.G.); laura.rodriguezmartin@vito.be (L.R.M.); greet.schoeters@vito.be (G.S.); eva.govarts@vito.be (E.G.); 16 UK Health Security Agency, London SE1 8UG, UK; ovnair.sepai@phe.gov.uk; 17 Department of Hygiene, Medical University of Karakalpakstan, Uzbekistan, Nukus 230100, Uzbekistan

**Keywords:** acrylamide, glycidamide, exposure level, time-trend, HBM4EU

## Abstract

Acrylamide, a substance potentially carcinogenic in humans, represents a very prevalent contaminant in food and is also contained in tobacco smoke. Occupational exposure to higher concentrations of acrylamide was shown to induce neurotoxicity in humans. To minimize related risks for public health, it is vital to obtain data on the actual level of exposure in differently affected segments of the population. To achieve this aim, acrylamide has been added to the list of substances of concern to be investigated in the HBM4EU project, a European initiative to obtain biomonitoring data for a number of pollutants highly relevant for public health. This report summarizes the results obtained for acrylamide, with a focus on time-trends and recent exposure levels, obtained by HBM4EU as well as by associated studies in a total of seven European countries. Mean biomarker levels were compared by sampling year and time-trends were analyzed using linear regression models and an adequate statistical test. An increasing trend of acrylamide biomarker concentrations was found in children for the years 2014–2017, while in adults an overall increase in exposure was found to be not significant for the time period of observation (2000–2021). For smokers, represented by two studies and sampling for, over a total three years, no clear tendency was observed. In conclusion, samples from European countries indicate that average acrylamide exposure still exceeds suggested benchmark levels and may be of specific concern in children. More research is required to confirm trends of declining values observed in most recent years.

## 1. Introduction

Human Biomonitoring for the European Union (HBM4EU), https://www.hbm4eu.eu/about-us/ (accessed on 14 July 2022) [1], is a multinational scientific project with the aim of gaining knowledge about the internal concentration of specific pollutants and contaminants within the European population using human biomonitoring. Thus, HBM4EU aims to close gaps on knowledge about exposure to several substances of concern, including acrylamide, in European populations and to complement existing knowledge [2]. Among a number of validated biomarkers, urinary indicators of acrylamide exposure were selected because of the associated potential risks for public health.

Based on experiments in rodents, acrylamide was assigned as a possibly carcinogenic substance [3,4]. Several other adverse health effects were recognized in connection with acrylamide intake, including neurotoxicity [5,6] and impaired fertility [7]. Acrylamide represents a widespread contaminant in many dietary products as well as in cigarette smoke [8,9,10]. Individual smoking habits have been shown to largely determine the levels of acrylamide biomarkers [9,11,12,13].

Acrylamide is formed by the Maillard reaction, a non-enzymatic reaction occurring in heated food products containing sugar and amino acids [14], but is also found in products such as cereals [15], bakery products [16], dried fruits, olives [17] and coffee [18]. Acrylamide exposure has been observed to be age dependent using blood [19] and urine biomarkers [20,21], with higher levels in younger ages and lower in adults.

Mitigating the dangers arising from carcinogens is generally complicated by a comparatively long induction time, which blurs both causal relationships and the quantification of the correlation between exposure concentration and effect. To support the development of responsible health policies, it is therefore vital to gain knowledge about the actual levels as well as time-trends of exposure. Together with the existing guidance values, those findings could be used to assess future consequences for public health and subsequently provide the scientific base for potential measures to be imposed with the aim to reduce exposure and related health risks.

Acrylamide exposure can be quantified in individuals by biomarkers found in blood and urine. Within studies aligned with HBM4EU (participating studies having collaborated on aligning human biomonitoring studies in the general population with combined financing from countries and HBM4EU), the urinary levels of mercapturic acids of acrylamide (AAMA, N-acetyl-S-(carbamoylethyl)-l-cysteine) and its epoxide metabolite glycidamide (GAMA, N-acetyl-S-(1-carbamoyl-2-hydroxyethyl)-l-cysteine) were determined. AAMA and GAMA can be quantified by high-performance liquid chromatographic (HPLC) or gas chromatographic (GC) separation methods and subsequent mass spectrometry [22,23].

Despite the fact that glycidamide represents a reactive epoxide metabolite of acrylamide, both substances indicate different hallmarks in acrylamide-related risk assessment. While the acrylamide metabolite AAMA may be primarily seen as a marker for exposure, glycidamide is the major contributor to DNA-damage and associated cancer risk [24]. The formation of glycidamide from acrylamide requires metabolization by cytochrome P450 (CYP2E1) [25] and conjugation to glutathione (GSH) [26]. The regional distribution of polymorphisms affecting involved proteins may thus potentially result in differences in the efficiency of acrylamide metabolism [27,28]. CYP2E polymorphisms may thus contribute to observed regional differences in average GAMA concentrations.

The main aim of this paper was to explore the time-trends of acrylamide exposure based on biomonitoring samples obtained by HBM4EU-aligned studies (ESTEBAN, GerES V, ESB, Oriscav-Lux2, Diet-HBM, INSEF-ExpoQuim, NEB II and NAC II) and to describe recent levels of acrylamide biomarkers in sub-populations of several European countries. Thus, we here set out to investigate trends in the AAMA and GAMA levels of populations from different regions of Europe with a focus on children as a vulnerable population and smokers as a potentially highly exposed population.

Since the recognition of acrylamide as a potential carcinogenic in 2001 [29], the results of several independent European human biomonitoring studies have been published [8,12,13,19,21,30,31,32,33,34,35,36,37,38,39,40,41,42,43,44,45,46], often focusing on the acrylamide exposure of specific population segments and using different standards for sampling and evaluation. Our study represents the first approach to investigate acrylamide exposure levels by biomonitoring in Europe populations, based on samples collected by contributing multi-national institutions and using common standards for data sampling quality assurance and evaluation. Despite the fact that the biomarkers of exposure have not been collected in a sufficient number of regions to be representative for the total European population, the obtained large database allows for a first analysis of trends related to acrylamide exposure time development within several contributing European populations.

## 2. Materials and Methods

### 2.1. Data Sources

The European countries/studies providing acrylamide data were Italy (Section of Hygiene and Epidemiology within the Department of Medical and Biological Sciences of the University of Udine, EPIUD: Northern Adriatic cohort II, NAC); Portugal (National Institute of Health Dr. Ricardo Jorge, INSA: Exposure of the Portuguese Population to Environmental Chemicals: a study nested in INSEF, INSEF-ExpoQuim); Germany (German Environment Agency, UBA: German Environmental Survey 2014–2017, GerES V and Environmental Specimen Bank, ESB (ESB started to collect samples in 2000 and was 2017 assigned as an HBM4EU-aligned Study); France (Agence Nationale De Santé Publique, ANSP: Etude de santé sur l’environnement, la biosurveillance, l’activité physique et la nutrition, ESTEBAN); Luxembourg (Laboratoire national de santé, LNS: Observation of cardiovascular risk factors in Luxembourg and Luxembourg Institute of Health, LIH, Oriscav-Lux2); Iceland (University of Iceland, UI: Icelandic National Dietary Survey Diet-HBM) and Norway (Norwegian Institute of Public Health, NIPH: Norwegian Environmental Biobank II, NEB II). The Norwegian Environmental Biobank is a substudy within MoBa established with the aim of biomonitoring nutrients and environmental contaminants in mothers, fathers and children participating in MoBa. The study included approximately six hundred triads of mothers, fathers and children who donated blood and urine samples, and responded to a questionnaire. The key parameters of the contributing studies are described in the tables below (Table 1 and Table 2). GerES V and ESB (Germany) provided extended datasets for this study based on bilateral agreements. GerES V provided samples from children and teenagers, NEB II and EPIUD provided samples from children and ESTEBAN collected samples from children and adults. All other contributing studies collected data from adults only. The actual data characteristics are shown in the table below. The descriptive statistics of the studies are shown in Appendix B, Table A3, Table A4, Table A5, Table A6, Table A7, Table A8 and Table A9.

Individual concentrations of urinary exposure biomarkers are generally dependent on urinary dilution. To adjust for this, urinary creatinine, which is fairly independent of the urine water content, at constant glomerular filtration rates and normal kidney function, has also been measured in the urine samples [47]. Specific gravity, considered a reliable measure of urine dilution, was not consistently available in the datasets used for this analysis. Therefore, the AAMA and GAMA levels used in this study are reported in µg/g creatinine.

Included studies provided data for acrylamide biomarkers derived from adults (age 20–39 years) or children and teenagers (age 3–18 years) on an individual level. Studies were performed between the years 2000 and 2021 in specific geographical and demographic population segments. Thus, the results presented herein have to be understood as indicative samples and not generally representative for countries, regions or Europe (no country/population weights were applied, although GerES V was designed to be representative of the German population).

**Figure 1:**
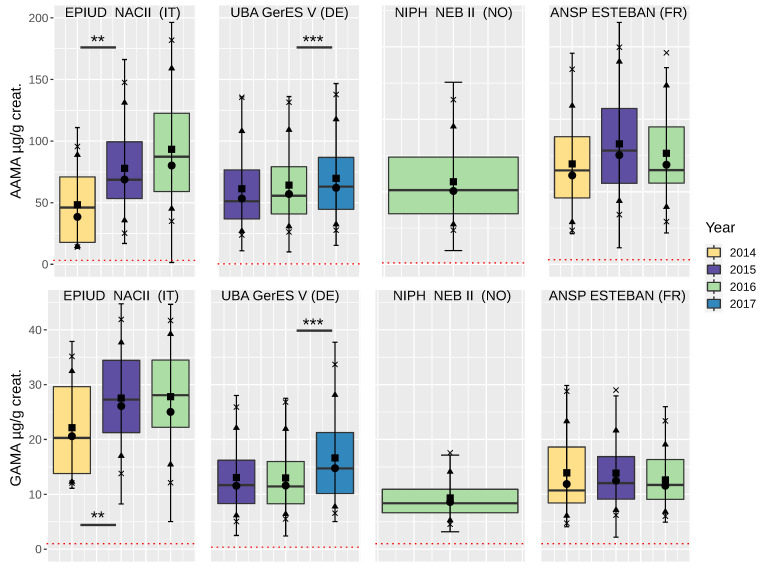
Boxplots of yearly mean (geom. mean, median) AAMA (top) and GAMA (bottom) concentrations in children and teenagers, based on data (non-smoker) of HBM4EU-aligned studies (Italy, NAC II; Germany GerES V; Norway, NEB II; and France, ESTEBAN). Box = 25–75% interquartile range; line = median; ■ = mean; ● = geometric mean; ▲ = 10 + 90% quantile; and x = 5 + 95% quantile. Dotted red line: level of quantification (LOQ). Asterisks indicate significant differences in (ln)AAMA or (ln)GAMA levels (one-way ANOVA), *** p<0.001, ** p<0.01.

**Figure 2:**
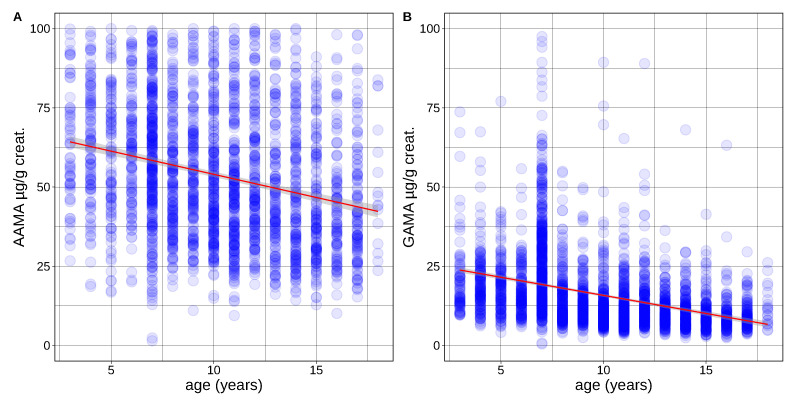
AAMA (**A**) and GAMA (**B**) (urine concentration in µg/g creatinine) in function of age in children and teenagers (3–18 years—from Germany (UBA, GerES V), France (ANSP, ESTEBAN) and Italy (EPIUD, NAC II). Linear fit in red, gray = 95% confidence interval.

The biomarker data were quality assured by the HBM4EU Quality Assurance/Quality Control program [48], see also Deliverable 9.4, The Quality Assurance/Quality Control Scheme in the HBM4EU project (https://www.hbm4eu.eu/work-packages/deliverable-9-4-the-quality-assurancequality-control-scheme-in-the-hbm4eu-project/ (accessed on 14 July 2022). In the applied QA/QC scheme for acrylamide, selected expert laboratories participated in three rounds of interlaboratory comparison investigations. The results were used to identify laboratories capable of generating consistent and comparable results for sample analysis in the frame of HBM4EU. Some datasets (ANSP ESTEBAN (children), UBA ESB, UBA GerES V, EPIUD NACII, NIPH NEBII) were generated before the establishment of the HBM4EU QA/QC program and comparability is therefore not guaranteed by the HBM4EU Quality Assurance Unit (QAU). The level of detection (LOD) was not provided by all studies. The level of quantification (LOQ) was found to be variable among the study groups and is therefore indicated in graphs (Figure 1 and Figure 2). Single values below LOQ were replaced by imputed random values taken between 0 and the limit as based on a determined lognormal distribution for this data segment. The number of samples below LOQ varied between datasets, ranging from 0% to a maximum of 8.11%.

First morning urine concentrations for AAMA and GAMA were reported by ESTEBAN (FRa+c), INSEF-ExpoQuim (PT) and GerES V (DE) (a very small number of samples from GerES V was collected too early or late and is thus considered spot urine). Spot urine was sampled by NAC II (IT), NEB II (NO), Diet-HBM (IS), and Oriscav-Lux2 (LU) and 24 h urine by ESB (DE). Differences in urine density (i.e., lower density in 24 h-samples compared to first morning and spot urine) as a consequence of these distinct sampling methods are considered not relevant in this analysis as these are based on creatinine-corrected concentrations.

### 2.2. Stratification

The main provided characteristics of participants that were anticipated to have an impact on biomarker concentrations were the age at time of sampling, smoking habits and year of sampling. As the determination of time-trends in AAMA and GAMA levels within single study populations was one of the main aims of this investigation, we stratified the data for age and smoking behavior.

#### 2.2.1. Age

As HBM4EU-aligned studies were performed in specific age groups by design, age strata are defined by given study populations and thus most countries are represented by either a population of children or adults (Table 1 and Table 2), with the exception of Germany and France, providing data from both age groups (ESTEBAN, GerES V and ESB). For direct comparisons of exposure levels, age groups were thus indicated and age was further used as a confounding variable in multivariate regression analysis.

#### 2.2.2. Smoking

We were able to stratify for non-smokers and smokers in studies performed in adults that were providing a sufficient number of smoking individuals. This was the case for data from ESTEBAN (FRa) and INSEF-ExpoQuim (PT). Small numbers of smokers in other studies were omitted in regio-temporal analysis, but included in overall smoker/non-smoker statistics.

### 2.3. Statistics

Statistical calculations were performed using R (R: A Language and Environment for Statistical Computing, R Core Team, R Foundation for Statistical Computing, Vienna, Austria, 2021, https://www.R-project.org/ (accessed on 14 July 2022)). If the sample number per year and study was below 20, it was not considered in descriptive comparisons on a yearly level, but included in the overall analysis. Data distribution was inspected for each dataset using frequency histograms (r-function hist) and by Q-Q-plots (function qqnorm and qqline, package stats, Version 3.6.2). Individual AAMA and GAMA levels (non-log-transformed) in µg/g creatinine did not show normal distribution in any dataset. Thus, for parametric statistical tests (including linear models, ANOVA), log-transformed values were used (using natural logarithm, ln) that have been shown to be normally distributed using the methods described above. Graphical depictions of according linear trends are shown using a non-log-transformed scale to allow for visualization of slopes at an original scale. The collinearity of the independent variables in multiple regression, which was anticipated due to the study design, was tested by the determination of a variance inflation factor (VIF, r-function vif, package regclass Version 1.6). A value of VIF < 1 was considered low collinearity; 1 ≤ VIF ≤ 5 was considered moderate collinearity; VIV > 5 was considered strong collinearity. Variables were not included in multiple regression if VIF was found to be >5 (this was only the case for dummy-variables indicating the individual studies and expected because of the predefined age range of participants in each study). Multiple regression was used for analyzing trends in pools containing data from more than one study/region, for the consideration of confounding variables associated with study-specific characteristics (age of participants, year of sampling). The geometric mean was calculated using the function gm_mean of the r-package tbrf (Version 0.15). For linear models, homoscedasticity was checked by residual plots and the Breusch–Pagan test (function bptest, package lmtest (Version 0.9–39). Means (after log transformation) were compared by ANOVA (function aov to generate a fit and subsequent function anova to test the generated fit) and the Tukey post hoc test (function TukeyHSD).

## 3. Results

### 3.1. Trends in Data-Pools of Non-Smokers and Detected Multicollinearity

We performed a multiple linear regression analysis for time-trends on data from 2000 to 2021 and 4187 samples of all non-smokers, under consideration of age and a categorical dummy variable for the sampling studies. Using this statistical method, a trend in (ln)AAMA and (ln)GAMA in µg/g creatinine over the time period of observation was found to be not significant (AAMA: p=0.371, GAMA: p=0.051), while age and study identifiers (dummy variables identifying the individual studies) were found to be significantly correlated (age, study ID, AAMA + GAMA: *p* < 0.001). However, as the given study design links specific age groups with study populations as well as to the years of sampling, we detected a high degree of multi-collinearity for the study identifier (i.e., country identifier). We thus further applied a strategy combining stratification and multiple linear regressions to avoid multi-collinearity.

### 3.2. Children and Teenagers (3–18 Years)

Acrylamide exposure, as mainly indicated by urine GAMA concentrations, was found to be higher in children from Italy (EPIUD, NAC II) compared to Germany (UBA, GerES V) Norway (NIPH, NEB II) and France (ANSP, ESTEBAN) (*t*-test log-data: AAMA, EPIUD vs. GerES V: p<0001, EPIUD vs. NEB II: p=0.0001, GAMA, EPIUD vs. GerES V: p<0.0001, EPIUD vs. NEB II: p<0.0001, EPIUD vs. ESTEBAN: p=0.0028, Figure 1) for the year 2016. Descriptive statistics are shown in Appendix B, Table A6. Direct comparison of (geometric) means between study populations is, however, not warranted due to partially overlapping sampling time periods and different mean population ages.

More conclusive are the comparisons of time-trends within studies with rather homogeneous populations. Median/geometric mean concentrations of AAMA and GAMA in Germany and Italy, with mean participant ages between 7.0 years and 10.3 years, show an increasing trend between 2014 and 2017 (Figure 1). The trend was stronger in the dataset from Italy than in Germany, but statistically significant in both datasets. The analysis of differences between single years (ANOVA and post hoc test) revealed significant differences for (ln)AAMA between 2014 and 2015, (p<0.006) in data from Italy and between 2016 and 2017 (p<0.0001) in samples from Germany. Accordingly, significantly different concentrations of (ln)GAMA were observed in samples from Italy between the years 2014 and 2015 (p<0.02) and in Germany between 2016 and 2017 (p<0.0001). For children from Norway, sufficient data were only available from one year. To summarize shortly, we see tendencies of rising exposure in children and teenagers in Germany and Italy and higher GAMA levels in Italy. An increasing trend was not observed in children from France (Appendix A, Table A1 Figure A2).

A comparison between 2807 individual children and teenagers (< 19 years) and 1091 adults (>18 years) revealed significantly higher levels of in (ln)AAMA and (ln)GAMA (µg/g creatinine) (AAMA: p<0.0001; GAMA: p<0.0001) in children and teenagers as compared to adults (log-transformed data, homogeneous variances, two-sample *t*-test).

To evaluate the impact of age on the measured levels of acrylamide biomarkers, multiple regression analysis was used to assess the association between acrylamide biomarker concentrations and age at the day of sampling using the individual data per cohort. The analyses revealed a high correlation for both AAMA and GAMA concentrations with age in children from Germany (GerES V), France (ESTEBAN) and Italy (NAC II) (Figure 2, Table 3). Higher biomarker levels were found at younger age groups.

The observed trend of lower exposure values in individual samples from older juveniles is in accordance with the finding of higher levels of exposure in children and teenagers compared to adults obtained using aggregated data.

### 3.3. Non-Smoking Adults (20–39 Years)

Within the different observation periods of the studies, the lowest levels for AAMA (in µg/creatinine) were found in adult non-smoking populations from Luxembourg (Oriscav-Lux2) and Germany (ESB) and slightly higher in Iceland (Diet-HBM), France (ESTEBAN) and Portugal (INSEF-ExpoQuim). GAMA levels are observed to be highest in samples from Portugal (INSEF-ExpoQuim). Again, time periods and age distribution were found to be different in each study population and the conclusiveness of direct comparisons between regions is limited.

**Figure 3:**
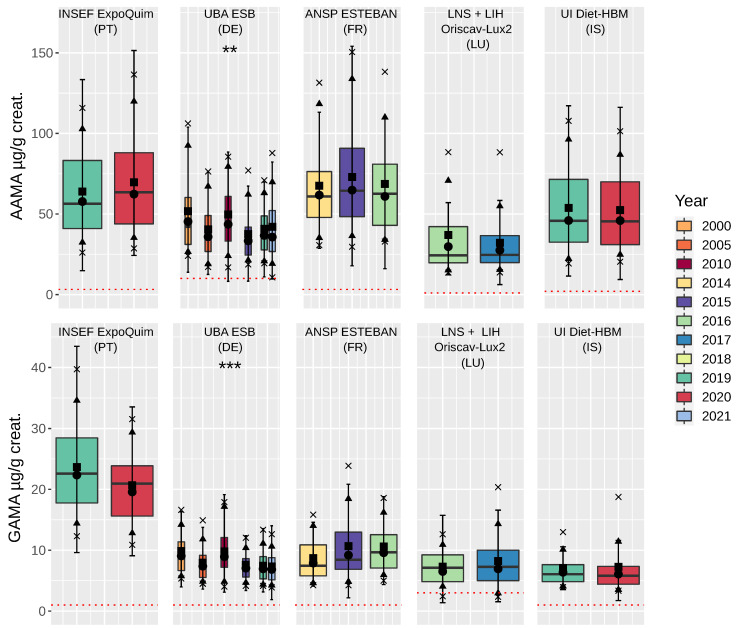
Boxplots of yearly mean (geom. mean, median) AAMA (top) and GAMA (bottom) in adults, based on data (non-smoker) of HBM4EU-aligned studies (Portugal, INSEF-ExpoQuim; Germany ESB; France, ESTEBAN; Luxembourg, LNS + LIH Oriscav-Lux2; and Iceland, Diet-HBM). Box = 25–75% interquartile range; line = median; ■ = mean; ● = geometric mean; ▲ = 10 + 90% quantile; and x = 5 + 95% quantile. Dotted red line: level of quantification (LOQ). Asterisks indicate significant differences in (ln)AAMA and (ln)GAMA in µg/g creatinine (one-way ANOVA), *** p<0.001, ** p<0.01.

An increasing time-trend between 2014 and 2017, as observed in children and teenagers, was not visible in adults (Figure 3). On the contrary, data from ESB show an overall trend of significantly declining concentrations between 2000 and 2021 (one-way ANOVA: (ln)AAMA µg/g creatinine: p=0.00454; (ln)GAMA µg/g creat: p<0.0001). The most prominent differences were found when comparing the data from 2015 with 2000 (p<0.014) and from 2015 with 2010 (p<0.05) in samples from ESB. Descriptive statistics are shown in Appendix B, Table A7 and Table A8.

Relatively stable or even declining biomarker levels within the sampling period for adults were also observed when evaluating individual data based on the sampling day instead of sampling year (see Appendix A, Table A2, Figure A3). A significant reduction over time was found in the data from Portugal, INSEF-ExpoQuim for GAMA, and for AAMA and GAMA in data from Germany, ESB.

**Figure 4:**
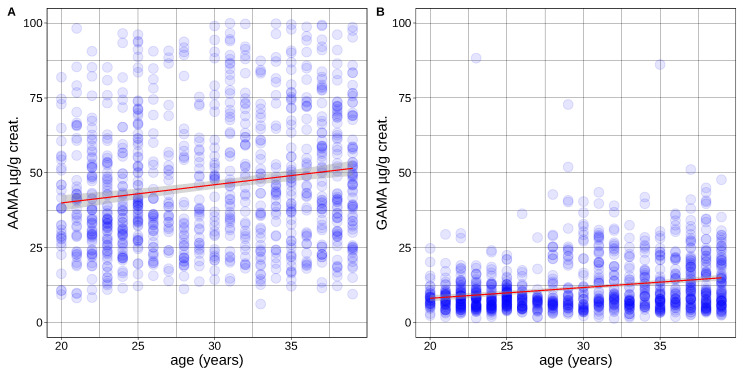
AAMA (**A**) and GAMA (**B**) (urine concentrations in µg/g creatinine) in function of age in non-smoking adults (20–39 years, Germany ESB; Luxembourg, LNS + LIH Oriscav-Lux2; Iceland, Diet-HBM; France, ESTEBAN; and Portugal, INSEF-ExpoQuim). Linear fit in red, gray = 95% confidence interval.

In multiple linear regression analyses, GAMA and AAMA urine concentrations were found to correlate with age in adults, with slightly higher levels observed at older ages (Table 4). The correlation between acrylamide biomarker concentrations and the age of the subjects is also illustrated in Figure 4. In total, considering the findings in children and teenagers, we observe a clear tendency of the lower exposure marker levels of AAMA and GAMA in older juveniles followed by a weak increase with age in adults.

### 3.4. Smoking Adults (20–39 Years)

Smokers are represented by a comparably small number of only 174 participants from two studies. Mean AAMA and GAMA levels (in µg/g creatinine) were found to be significantly higher in smokers as compared to non-smokers. A summarized comparison of 174 smoking and 1091 non-smoking adults revealed significantly higher levels of in AAMA and GAMA (µg/g creatinine) (AAMA: p<0.0001, GAMA: p<0.0001) in smokers.

**Figure A1:**
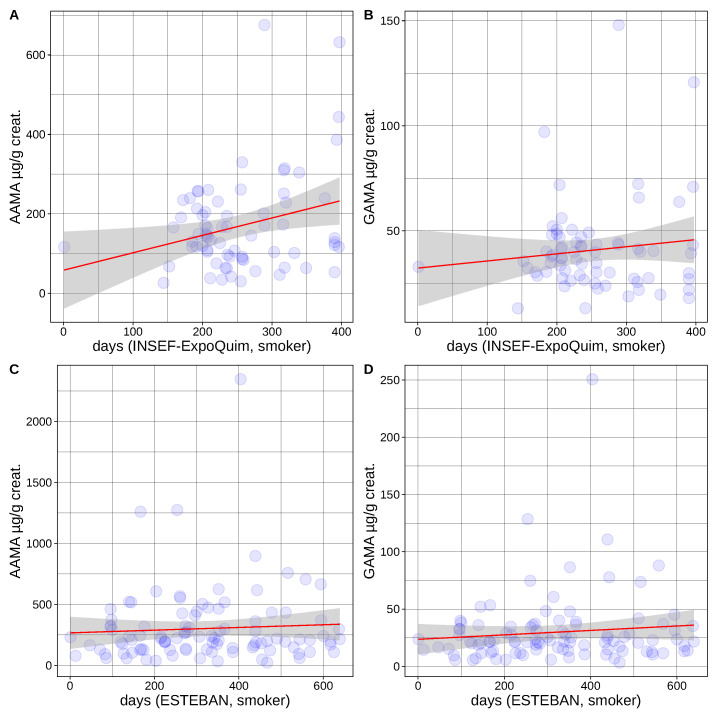
Time-trend of AAMA (**A**,**C**) and GAMA (**B**,**D**) urine concentrations in µg/g creatinine (per sampling day, 0 = first day of sampling) in individual data (smoker) from HBM4EU-aligned studies from Portugal (INSEF-ExpoQuim, top) and France (ESTEBAN, bottom). Linear fit in red, gray = 95%. Trends were found to be not significant in statistical analysis.

**Figure A2:**
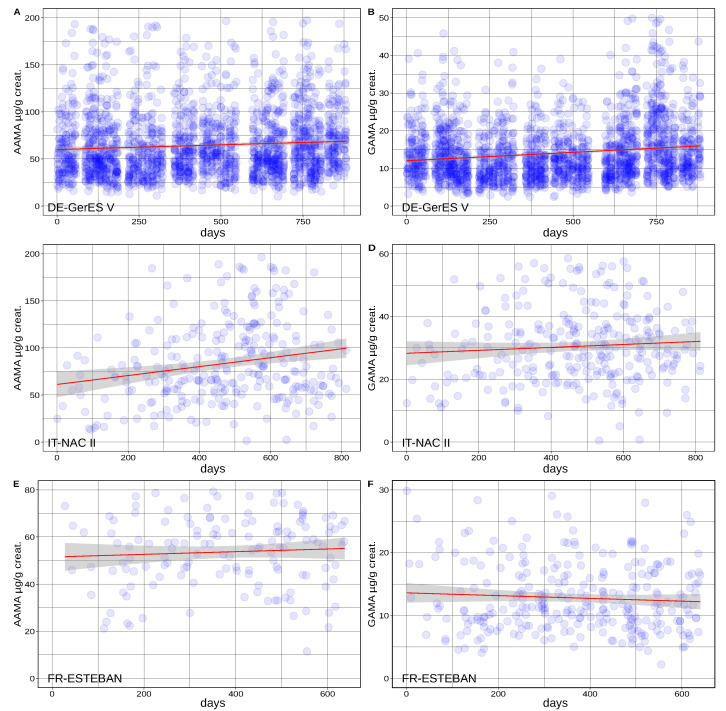
Time-trend of AAMA and GAMA urine concentrations in µg/g creatinine (per sampling day, 0 = first day of sampling) in individual data (non-smoker, children and teenagers) HBM4EU aligned studies from Germany (UBA, GerES V, **A**,**B**), Italy (EUPID, NAC II, **C**,**D**) and France (ESTEBAN, **E**,**F**). Linear fit in red, gray = 95% confidence interval.

**Figure A3:**
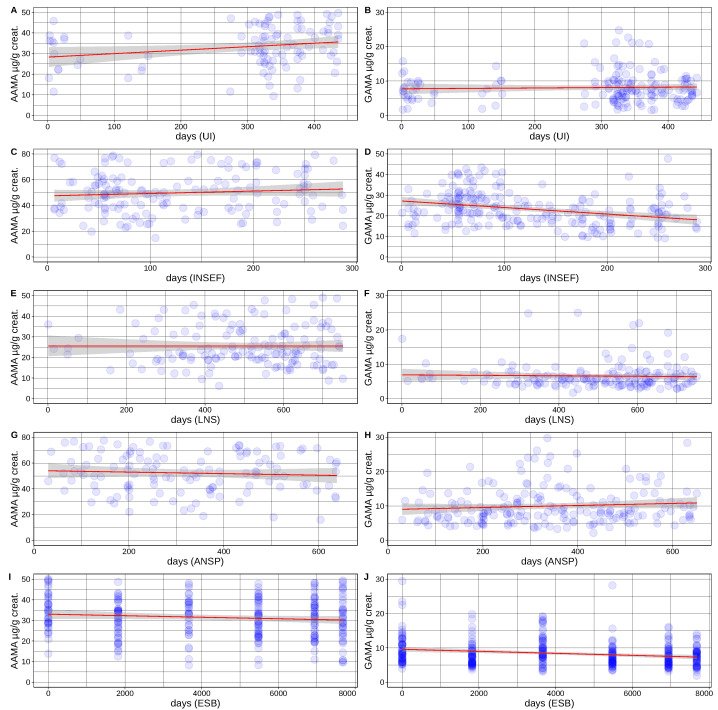
Time-trend of AAMA (**A**,**C**,**E**,**G**,**I**) and GAMA (**B**,**D**,**F**,**H**,**J**) in µg/g creatinine (per sampling day, 0 = first day of sampling) in individual data (non-smoker, adults) from HBM4EU-aligned studies from Iceland (Diet-HBM), Portugal (INSEF-ExpoQuim), Luxembourg (Oriscav-Lux2), France (ESTEBAN) and Germany (ESB). Linear fit in red, gray = 95% confidence interval.

Due to low sample numbers, a comparison of yearly medians/geom. means is not conclusive for smokers. Descriptive statistics of studies are shown in Appendix B, Table A9. Time-trends in smoking adults were analyzed using available individual data from Portugal (ExpoQuim) and France (ESTEBAN) (Appendix A, Figure A1). Regression analysis using a linear model (after normalization by logarithmic transformation using natural logarithm, ln) did not reveal a significant time-trend in individual data from smokers.

## 4. Discussion

Based on our results, the means of current biomarker samples from Europe are expected to exceed the biomonitoring equivalent (BE) for acrylamide which was established at 16 µg/g creatinine for AAMA (for an averagely aged population). BE values are proposed as an interim solution for the determination of a safe margin of exposure, while epidemiological surveys providing health guidance values for acrylamide have not been established yet. This value has been calculated for different age groups (children < 13 years, adolescents 13–18 years, adults >19 years) based on doses determined in animal experiments [49] and on a US risk assessment (USEPA, 2007b) [50] which concluded that the area under the serum curves (AUC) for acrylamide and glycidamide represents the appropriate dose metrics for neurological and tumor responses. However, as risk-specific doses and risk levels for cancer and non-cancer endpoints differ in magnitude, a high level of uncertainty remains within common acrylamide BE value estimates. The European HBM-guidance values for acrylamide therefore need to be updated in the near future, based on risk assessments in 2015 and 2022 [2,51].

For children below the age of 13 years, a BE of 20 µg/g creatinine was calculated and for men and women older than 19 years, a value of 15 µg/g creatinine (AAMA). These levels are, according to our results, only met/unattained by the low 10% quantile of samples from Luxembourg (q10 = 15.46, adults, 2016–2018). With geometric mean values of 73.17 µg/g creatinine for AAMA, data from France (ANSP, ESTEBAN) showed the highest value for non-smoking adults and data from Italy (EPIUD, NAC II) showed the highest value for children with a geometric mean of 78.58 µg/g creatinine, indicating biomarker levels that were 4 to 5 times higher than the suggested BE values and in accordance with previously reported values [38].

Even much higher values were found in smokers with geometric means of 135.92 µg/g creatinine for AAMA in Portugal (INSEF-ExpoQuim) and 218.98 µg/g creatinine in France (ESTEBAN). Data from Portugal show ∼2 times the geometric mean found in non-smokers of the same population (60.8 µg/g creatinine) and data from France (73.17 µg/g creatinine) ∼3 times. This is well in line with exposure levels reported for smokers by other European studies [12,13,30,46]. Acrylamide inhalation by smoking represents a very different form of exposure, as compared to dietary intake and may result in a different related cancer risk. A physiologically based toxicokinetic (PBTK) model [52] comparing inhalative intake to oral exposure of acrylamide revealed, however, that both forms of intake may result in a very similar cancer risk in relation to equivalent doses [53].

Our results indicate higher levels and larger differences in the biomarker levels of acrylamide in children compared to adults and are therefore in accordance with the results by U. Heudorf [38]. Vesper et al. [54] did not find higher blood adduct levels in US children, while Hartmann et al. [21] found higher levels in teenagers compared to adults in blood adducts and urine biomarkers.

As most studies were performed in populations of predefined age ranges, specific regional trends may be represented to a higher degree in the according age groups. However, we have reason to believe that the higher observed acrylamide biomarker levels in children as compared to adults are indeed related to the age and not due to region-specific confounding variables, as (i) levels reported for adults and children/adolescents in the German and French studies (ESTEBAN, ESB and GerES V), with overlapping sampling periods showed higher levels in children; and (ii) results from studies comprising participants of different age show a significant age dependence of acrylamide biomarkers within the same study population.

Increased levels observed in children may be due to a higher intake in this population segment. There are published exposure assessments supporting this hypothesis, including an FAO/WHO report, indicating a dietary acrylamide intake in children that is two-to-three times higher than those of adults [55,56].

A possible higher intake in children may coincide with a reduced detoxification potential, resulting in overall higher tissue concentrations. This has been proposed in a PBTK model introduced by Walker et al., 2007 [57], where the enzyme activity of an immature physiology was considered in an explorative toxicokinetic model of acrylamide metabolism. The authors concluded that the estimated elevations in glycidamide area-under-the-curve (AUC) in children may lead to increased tissue binding and, in combination with a higher sensitivity to mutagenic chemicals in early life [58], to affect cancer risk estimates in children as compared to adults. Results from experiments in rodents indicate a neurotoxic effect of acrylamide for the developing brain, adding a further potential risk related to acrylamide exposure in early life [59,60,61]. In combination, these results emphasize once again the need for specific attention to younger ages with regard to acrylamide-related health risks. In this context, our finding that acrylamide biomarker levels were increasing between 2014 and 2017 in the populations representing children is worrisome. Limitations of provided datasets imply that children and adolescents were only represented by three regional study groups, one not allowing for a time-trend analysis due to the data structure and provided parameters, and no data from Eastern Europe were obtained. However, because we were able to include data provided by GerES V, the presented trend is based on a large total number of participants. Data from GerES V on children and adolescents have already been analyzed in detail, summarized and presented in a study-dedicated publication [46]. It is possible, however, that the observed trends are not present in other regions and populations. Differences of the mean acrylamide biomarker observed between regions/studies may be due to specific regional intake levels, but, at least for GAMA, may also be explained by regional differences in prevalence to cytochrome P450 (CYP2E1) polymorphisms [62]. Furthermore, we have no information if the time-trend in children continues after 2017, as included studies sampling at later time points did focus on adult populations.

As high exposure levels and an increasing tendency of acrylamide biomarkers levels are found in children and teenagers, representing a very vulnerable population segment with regard to cancer risk, comprehensive studies performing the human biomonitoring of acrylamide biomarkers in Europe should continue to allow the validation of findings, the consideration of recent developments and, if required, the adjustment of mitigation measures.

## Figures and Tables

**Table 1:** Overview of HBM4EU-aligned studies and data sources based on bilateral agreements performing biomonitoring acrylamide metabolites, performed between 2014 and 2017 in teenagers and children.

Provider of Data	Study Label	Data Code	Year of Sampling	Number of Participants (Non-Smoker)	Mean Age (Years)	Age Range
EPIUD	NAC II	IT1	2014	18	7.0	7
EPIUD	NAC II	IT2	2015	132	7.2	7–8
EPIUD	NAC II	IT3	2016	147	7.0	7
UBA	GerES V	DE1	2015	852	10.3	3–18
UBA	GerES V	DE2	2016	849	10.3	3–18
UBA	GerES V	DE3	2017	517	10.3	3–18
NIPH	NEB II	NO	2016	289	9.8	7–11
ANSP	ESTEBAN	FR1c	2014	55	8.5	6–11
ANSP	ESTEBAN	FR2c	2015	208	8.9	6–11
ANSP	ESTEBAN	FR3c	2016	37	8.9	6–11

**Table 2:** Overview of HBM4EU-aligned studies and data sources based on bilateral agreements performing biomonitoring acrylamide metabolites, performed between 2000 and 2021 in adults.

Provider of Data	Study Label	Data Code	Year of Sampling	Number of Participants (Non-Smoker)	Number Participants (Smoker)	Mean Age(Years)	Age Range
UI	Diet-HBM	IS1	2019	289	6	31.6	21–39
UI	Diet-HBM	IS1	2020	154	12	30.6	20–39
INSA	INSEF-ExpoQuim	PT1	2019	177	67	34.5	28–39
INSA	INSEF-ExpoQuim	PT2	2020	37	12	34.7	28–39
LNS+LIH	Oriscav-Lux2	LU1	2016	34	7	33.3	26–39
LNS+LIH	Oriscav-Lux2	LU2	2017	123	25	33.5	25–39
LNS+LIH	Oriscav-Lux2	LU3	2018	12		36.0	33–39
UBA	ESB	ESB1	2000	60		24,4	20–29
UBA	ESB	ESB2	2005	60		23.6	20–28
UBA	ESB	ESB3	2010	60		23.3	20–28
UBA	ESB	ESB4	2015	60		23.0	20–28
UBA	ESB	ESB5	2019	60		23.0	20–28
UBA	ESB	ESB6	2021	54		23.0	20.28
ANSP	ESTEBAN	FR1a	2014	36	27	31.4	20–39
ANSP	ESTEBAN	FR2a	2015	138	64	32.5	20–39
ANSP	ESTEBAN	FR3a	2016	23	10	34.0	26–39

**Table 3:** Estimated slope (s) and statistical significance of a multiple regression for AAMA and GAMA in µg/g creatinine (after normalization by logarithmic transformation using natural logarithm, ln) and age in years regression for AAMA and GAMA in µg/g creatinine and age in years in children and teenagers. ***: p<0.001, **: p<0.01.

Variable	AAMA (ln(µg/g Creat.)/Year)	GAMA(ln(µg/g Creat.)/Year)
Age (years)	s: −0.04, ***	s: −0.072, ***
Sampling year	s: 0.04, **	s: 0.061, ***

**Table 4:** Estimated slope (s) and statistical significance of a multiple linear regression for AAMA and GAMA in µg/g creatinine (after normalization by logarithmic transformation using natural logarithm, ln) and age in years in adults. ***: p<0.001, ns = not significant.

Variable	AAMA (ln[µg/g Cerat.]/Year)	GAMA (ln[µg/g cerat.]/Year)
Age (years)	s: 0.018, ***	s: 0.0239, ***
Sampling year	s: −0.004, ns	s: 0.0024, ns

## Data Availability

Data visualizations will be made available at https://www.hbm4eu.eu/what-we-do/european-hbm-platform/eu-hbm-dashboard/ (last access 14 July 2022).

## Abbreviations

The following abbreviations are used in this manuscript:

AAMA	N-acetyl-S-(carbamoylethyl)-l-cysteine)
GAMA	N-acetyl-S-(1-carbamoyl-2-hydroxyethyl)-l-cysteine)
HBM4EU	Human Biomonitoring for European Union
CYP2E1	Cytochrome P450 2E1

## Appendix A

**Table A1:** Estimated slope (s) and statistical significance of a multiple linear regression for AAMA and GAMA in µg/g creatinine (after normalization by logarithmic transformation using natural logarithm, ln) and in function of the sampling day in children. ***: p<0.001; ns: not significant.

Study	AAMA (ln(µg/g Creatinine)/Day)	GAMA(ln(µg/g Creatinine)/Day)
UBA, GerES V (Germany), children	s: 0.0002, ***	s: 0.0003, ***
EPIUD, NAC II (Italy), children	s: 0.0008, ***	s: 0.0003, ns
ANSP, ESTEBAN (France), children	s: 0.0004, ns	s: −0.0003, ns

**Table A2:** Estimated slope (s) and statistical significance of a multiple linear regression for AAMA and GAMA in µg/g creatinine (after normalization by logarithmic transformation using natural logarithm, ln) and in function of the sampling day in adults. ***: p<0.001, **: p<0.01, ns: not significant.

Study	AAMA (ln(µg/g Creatinine)/Day)	GAMA (ln(µg/g Creatinine)/Day)
UI (Diet-HBM, Iceland)	s: 0.0003, ns	s: 0.0001, ns
INSEF (ExpoQuim, Portugal)	s: 0.0005, ns	s: −0.0013, ***
LNS + LIH (Oriscav-Lux2, Luxembourg)	s: 0.00001, ns	s: −0.0001, ns
ANSP (ESTEBAN, France)	s: 0.0005, ns	s: 0.0005, ns
UBA (ESB, Germany)	s: −0.00003, **	s: −0.00003, ***

## Appendix B

The following Table A3, Table A4, Table A5, Table A6, Table A7, Table A8 and Table A9 summarize descriptive statistics for the studies included in this analysis. Urine concentrations are provided in µg/L (AAMA and GAMA) and in µg/g creatinine (AAMA-crt and GAMA-crt). AGE = descriptive statistics of population age; study = sampling institution; pop = number of samples; type = reported value; mean = mean value; sd = standard deviation; geom. mean = geometric mean; min = minimal value; max = maximal value; median = median; and q10–q90 = quantiles 10–90%.

**Table A3:** Descriptive statistics of acrylamide biomarker levels per study (non-smoker I).

Study	Pop	Type	Mean	Sd	Geom. Mean	Min	Max	Median	q10	q25	q75	q90
IS-UI	171	AAMA-crt	59.7	52.7	48.39	9.36	521.5	45.83	24.08	31.11	71.76	96.15
IS-UI	171	AAMA	80.92	68.97	61.47	7.85	620.58	60.17	24.97	35.11	108.15	154.35
IS-UI	171	GAMA-crt	8.48	5.76	7.15	1.53	51.93	7.28	3.37	5.34	9.96	14.27
IS-UI	171	GAMA	12.13	9.68	8.84	1.5	61.8	9.97	3.13	5.13	15.55	23.03
IS-UI	171	AGE	30.63	5.37	30.13	20	39	30	23	26	35	38
DE-ESB	354	AAMA-crt	43.75	24.67	38.21	8.19	148.21	36.51	21.46	28.17	52.94	74.31
DE-ESB	354	AAMA	37.56	31.19	28.54	5	250	28.4	11.66	17.43	46.78	72.84
DE-ESB	354	GAMA-crt	8.35	3.76	7.67	1.89	29.44	7.66	4.6	5.8	9.75	12.85
DE-ESB	354	GAMA	7	4.99	5.73	0.5	38.1	5.95	2.6	3.8	8.3	12.57
DE-ESB	354	AGE	23.42	2.13	23.32	20	29	23	21	22	25	26
PT-INSEF	212	AAMA-crt	69.59	40.49	60.8	14.78	281.82	58.09	33.34	41.89	85.89	113.85
PT-INSEF	212	AAMA	84.27	60.47	67.59	7.5	347.67	66.72	28.45	44.82	108.61	162.61
PT-INSEF	212	GAMA-crt	23.79	9.58	22.2	9.09	86.16	22.38	13.8	17.37	28.31	34.62
PT-INSEF	212	GAMA	28.88	16.51	24.68	5.39	114.04	25.17	12.23	16.63	37.57	52.23
PT-INSEF	212	AGE	34.69	3.35	34.52	28	39	35	30	32	38	39
LU-LNS+LIH	157	AAMA-crt	35.58	38.44	28.48	6.17	413.73	24.45	15.46	19.79	39.11	56.77
LU-LNS+LIH	157	AAMA	69.3	81.45	47.7	4.2	730.1	49	17.32	25.8	78.3	132.54
LU-LNS+LIH	157	GAMA-crt	7.22	5.82	6.15	1.73	43.5	5.84	3.64	4.45	7.41	10.89
LU-LNS+LIH	157	GAMA	14.2	15.72	10.29	1.4	136.8	10.6	3.66	6.4	16	23.16
LU-LNS+LIH	157	AGE	33.54	3.82	33.32	25	39	33	28	31	37	38.4

**Table A4:** Descriptive statistics of acrylamide biomarker levels per study (non-smoker II).

Study	Pop	Type	Mean	Sd	Geom. Mean	Min	Max	Median	q10	q25	q75	q90
NO-NIPH	289	AAMA-crt	63.61	57.1	53.08	9.44	801.62	52.14	27.59	35.02	75.72	102.5
NO-NIPH	289	AAMA	75.92	105.85	56.6	8.1	1615	53.7	25.96	35.1	84.8	135.88
NO-NIPH	289	GAMA-crt	9.54	5.33	8.66	3.17	65.32	8.38	5.28	6.64	10.96	14.28
NO-NIPH	289	GAMA	11.13	9.95	9.24	1.7	131.6	8.9	4.68	6.7	12.6	18.62
NO-NIPH	289	AGE	9.82	1.17	9.74	7	11	10	8	9	11	11
DE-GerES V	2218	AAMA-crt	73.03	63.3	59.93	10.11	1000	56.93	30.03	40.05	83.63	125.61
DE-GerES V	2218	AAMA	92.56	89.15	70.05	2.8	1490	70.05	28.47	45.23	109	171
DE-GerES V	2218	GAMA-crt	14.53	9.51	12.51	2.42	147.01	12.21	6.52	8.62	17.55	24.72
DE-GerES V	2218	GAMA	17.83	12.23	14.62	0.5	130	15	6.5	10	22.3	31.63
DE-GerES V	2218	AGE	10.3	4.08	9.35	3	18	11	4	7	14	16
IT-EPIUD	300	AAMA-crt	100.24	94.61	78.58	1.46	993.34	78.68	37.97	55.76	119.37	180.35
IT-EPIUD	300	AAMA	88.59	73.93	66.03	1.6	757.02	72.51	22.22	46.48	109.65	160.86
IT-EPIUD	300	GAMA-crt	34.54	19.8	29.9	0.46	174.84	30.74	16.65	22.36	39.94	55.41
IT-EPIUD	300	GAMA	30.89	20.17	25.13	0.5	174.66	27.11	10.15	17.86	38.93	54.4
IT-EPIUD	300	AGE	7.02	0.18	7.02	6	8	7	7	7	7	7
FR-ESTEBAN	197	AAMA-crt	90.51	70.95	73.17	16.02	493.12	68.79	36.76	49.25	108.99	168.24
FR-ESTEBAN	197	AAMA	83.1	70.99	65.62	5.73	588.88	67.4	28.56	42.51	101.59	148.16
FR-ESTEBAN	197	GAMA-crt	10.91	8.72	9.17	2.18	88.3	8.53	4.86	6.5	12.84	18.45
FR-ESTEBAN	197	GAMA	10.11	7.87	8.22	0.5	69.96	8.5	3.53	5.64	12.77	17.47
FR-ESTEBAN	197	AGE	32.72	5.23	32.26	20	39	34	25	29	37	39

**Table A5:** Descriptive statistics of acrylamide biomarker levels per study (smoker).

Study	Pop	Sype	Mean	Sd	Geom. Mean	Min	Max	Median	q10	q25	q75	q90
PT-INSEF	72	AAMA-crt	168.58	119.23	135.92	26.38	675.61	140.08	56.39	93.83	217.21	299.83
PT-INSEF	72	AAMA	228.03	187.44	164.47	29.3	893.75	175.18	47.36	95.81	293.13	498.81
PT-INSEF	71	GAMA-crt	40.87	21.73	36.91	13.09	148	37.17	22.53	28.08	44.33	63.78
PT-INSEF	71	GAMA	52.04	30.52	44.22	13.55	170.69	43.53	19.71	29.46	66.36	92.78
PT-INSEF	72	AGE	34.11	2.96	33.98	28	39	34	30	32	36	38
FR-ESTEBAN	102	AAMA-crt	302.29	302.67	218.98	22.98	2346.83	217.83	81.12	131.47	367.95	565.61
FR-ESTEBAN	102	AAMA	288.69	202.08	225.61	39.78	959.76	227.42	81.44	143	396.5	548.49
FR-ESTEBAN	102	GAMA-crt	29.79	30.68	22.32	3.25	250.68	21.7	9.8	13.99	34.46	51.66
FR-ESTEBAN	102	GAMA	27.62	16.66	23	5.63	115.89	25.63	9.36	14.42	38.28	47.58
FR-ESTEBAN	102	AGE	32.38	4.78	32.01	20	39	33	26	29	36	38

**Table A6:** Descriptive statistics of acrylamide biomarker levels per study and year (children and teenagers).

Study	Pop	Year	Sample	Mean	SD	Geom. Mean	Min	Max	Median	q10	q25	q75	q90
FR-ESTEBAN	55	2014	AAMA	103.36	74.22	81.15	21.14	349.44	77.24	35.05	48.13	141.23	213.73
FR-ESTEBAN	207	2015	AAMA	115.66	130.22	89.05	11.43	1309.23	84.65	44.26	57.86	126.99	191.81
FR-ESTEBAN	37	2016	AAMA	89.47	50.36	76.88	21.67	218.46	68.104	40.81	56.70	113.32	170.74
FR-ESTEBAN	55	2014	GAMA	13.93	8.53	11.89	4.08	44.85	10.71	6.10	8.44	18.65	23.32
FR-ESTEBAN	207	2015	GAMA	15.19	11.18	13.00	2.19	110.44	12.41	7.25	9.26	17.55	22.82
FR-ESTEBAN	37	2016	GAMA	12.64	5.30	11.58	4.92	25.98	11.72	6.82	9.09	16.36	19.10
NO-NEBII	289	2016	AAMA	63.61	57.10	53.08	9.44	801.62	52.14	27.59	35.02	75.72	102.50
NO-NEBII	289	2016	GAMA	9.54	5.33	8.66	3.17	65.32	8.38	5.28	6.64	10.96	14.28
IT-EPIUD	18	2014	AAMA	58.14	49.87	42.46	12.89	224.45	47.98	14.88	19.50	78.82	97.51
IT-EPIUD	133	2015	AAMA	91.12	92.77	73.63	16.90	992.60	70.34	36.48	54.22	109.00	146.45
IT-EPIUD	149	2016	AAMA	113.47	97.86	89.71	1.46	993.34	93.98	45.61	59.59	134.95	186.04
IT-EPIUD	18	2014	GAMA	23.80	10.52	21.67	11.12	51.85	20.88	12.31	14.79	29.91	35.51
IT-EPIUD	133	2015	GAMA	34.17	16.09	30.84	8.26	97.50	31.25	18.05	22.23	41.92	55.17
IT-EPIUD	149	2016	GAMA	36.16	22.99	30.24	0.46	174.84	30.94	16.89	23.37	40.05	56.45
DE-GerES V	852	2015	AAMA	67.52	57.27	55.60	11.04	780.10	51.61	27.23	37.24	78.91	115.60
DE-GerES V	849	2016	AAMA	69.68	53.51	59.07	10.11	774.44	56.24	30.88	41.31	82.01	121.66
DE-GerES V	517	2017	AAMA	87.63	82.46	69.44	15.31	1000.00	65.51	33.94	45.79	96.95	146.11
DE-GerES V	852	2015	GAMA	13.46	8.20	11.71	2.49	89.38	11.68	6.17	8.32	16.38	22.31
DE-GerES V	849	2016	GAMA	13.27	7.37	11.72	2.42	69.67	11.47	6.42	8.31	16.06	22.77
DE-GerES V	517	2017	GAMA	18.36	12.99	15.53	5.02	147.01	15.26	7.85	10.29	22.82	30.82

**Table A7:** Descriptive statistics of acrylamide biomarker levels per study and year (adults I).

Study	Pop	Year	Sample	Mean	SD	Geom. Mean	Min	Max	Median	q10	q25	q75	q90
DE-ESB	60	2000	AAMA	51.73	29.47	45.32	13.85	145.13	42.04	26.55	31.12	60.26	92.44
DE-ESB	60	2005	AAMA	40.43	21.62	35.85	12.50	131.74	34.61	18.97	26.70	49.04	67.15
DE-ESB	60	2010	AAMA	49.73	25.77	43.62	8.19	148.21	44.47	24.00	33.17	60.86	79.42
DE-ESB	60	2015	AAMA	37.51	20.40	33.37	8.34	132.29	32.57	21.33	24.50	41.90	62.11
DE-ESB	60	2019	AAMA	40.86	20.55	36.66	11.00	122.50	36.30	20.72	27.74	48.80	63.08
DE-ESB	54	2021	AAMA	42.08	25.64	35.67	9.63	144.29	34.83	19.16	26.71	52.13	69.79
DE-ESB	60	2000	GAMA	9.87	4.59	9.07	3.98	29.44	8.69	5.35	6.67	11.37	14.21
DE-ESB	60	2005	GAMA	7.95	3.37	7.38	3.61	19.82	7.28	4.85	5.52	9.17	11.81
DE-ESB	60	2010	GAMA	9.80	4.11	8.94	3.11	19.14	9.05	4.85	7.20	12.10	17.15
DE-ESB	60	2015	GAMA	7.60	3.57	7.06	3.39	28.20	6.89	4.67	5.61	8.63	10.39
DE-ESB	60	2019	GAMA	7.45	2.89	6.95	3.15	16.08	7.03	4.38	5.30	8.95	11.11
DE-ESB	54	2021	GAMA	7.35	2.64	6.85	1.89	14.02	7.39	4.25	5.14	8.79	10.62
IC-DietHBM	27	2019	AAMA	53.79	28.82	45.97	11.48	117.17	45.77	22.26	32.50	71.49	96.27
IC-DietHBM	144	2020	AAMA	60.81	55.99	48.86	9.36	521.50	45.94	25.26	31.14	71.57	95.50
IC-DietHBM	27	2019	GAMA	7.36	3.15	6.60	1.64	15.74	7.13	4.07	5.10	9.24	10.91
IC-DietHBM	144	2020	GAMA	8.68	6.10	7.26	1.53	51.93	7.38	3.20	5.35	10.11	14.35
POR-INSEF	175	2019	AAMA	69.56	41.75	60.48	14.78	281.82	57.97	32.90	41.54	85.35	112.82
POR-INSEF	37	2020	AAMA	69.71	33.90	62.32	24.29	159.59	63.47	35.30	43.88	88.02	119.92
POR-INSEF	175	2019	GAMA	24.30	9.83	22.68	9.62	86.16	22.70	14.43	17.80	28.68	35.59
POR-INSEF	37	2010	GAMA	21.39	7.90	20.05	9.09	47.70	21.22	12.93	15.77	23.95	30.02

**Table A8:** Descriptive statistics of acrylamide biomarker levels per study and year (adults II).

Study	Pop	Year	Sample	Mean	SD	Geom. Mean	Min	Max	Median	q10	q25	q75	q90
FR-ESTEBAN	36	2014	AAMA	70.72	34.64	63.64	28.74	182.77	61.75	35.65	48.59	76.94	126.40
FR-ESTEBAN	138	2015	AAMA	97.02	78.81	76.77	17.88	493.12	69.05	37.07	49.66	120.35	200.83
FR-ESTEBAN	23	2016	AAMA	82.40	55.11	68.23	16.02	257.73	69.79	35.03	43.25	95.25	141.77
FR-ESTEBAN	36	2014	GAMA	8.71	4.15	7.89	4.11	21.38	7.44	4.62	5.80	10.88	14.02
FR-ESTEBAN	138	2015	GAMA	11.54	9.89	9.47	2.18	88.30	8.51	4.85	6.88	13.30	20.40
FR-ESTEBAN	23	2016	GAMA	10.60	5.24	9.59	4.35	28.39	9.66	5.95	7.08	12.55	16.20
LU-LNS+LIH	34	2016	AAMA	37.01	30.90	29.75	12.23	169.78	24.30	15.14	19.71	42.14	70.68
LU-LNS+LIH	123	2017	AAMA	35.19	40.26	28.14	6.17	413.73	24.91	15.51	19.92	37.33	56.09
LU-LNS+LIH	34	2016	GAMA	7.07	4.06	6.38	3.54	24.82	6.07	4.09	4.85	7.64	10.11
LU-LNS+LIH	123	2017	GAMA	7.27	6.22	6.08	1.73	43.50	5.80	3.53	4.42	7.36	11.42
POR-INSEF	62	2019	AAMA	157.77	102.16	130.06	26.38	675.61	143.10	56.39	92.37	203.73	260.07
POR-INSEF	9	2020	AAMA	251.27	183.23	191.88	53.38	632.07	138.92	104.06	121.69	386.56	481.49
POR-INSEF	62	2019	GAMA	39.79	19.85	36.41	13.09	148.00	36.98	23.62	28.68	44.04	52.03
POR-INSEF	9	2020	GAMA	48.30	30.76	40.51	17.97	120.71	39.30	21.11	26.98	63.78	80.91

**Table A9:** Descriptive statistics of acrylamide biomarker levels per study and year (adults, smokers).

Study	Pop	Year	Sample	Mean	SD	Geom. Mean	Min	Max	Median	q10	q25	q75	q90
FR-ESTEBAN	27	2014	AAMA	255.54	238.11	189.25	46.65	1260.31	192.75	71.36	115.46	318.86	484.11
FR-ESTEBAN	64	2015	AAMA	322.55	337.86	227.84	22.98	2346.83	222.62	84.04	145.99	427.72	595.29
FR-ESTEBAN	11	2016	AAMA	299.19	196.00	248.76	108.99	706.27	216.37	110.05	183.06	333.85	665.53
FR-ESTEBAN	27	2014	GAMA	22.20	12.92	18.49	5.52	53.36	20.22	6.76	12.59	29.78	38.70
FR-ESTEBAN	64	2015	GAMA	32.84	36.34	23.60	3.25	250.68	22.36	10.26	14.78	34.96	69.73
FR-ESTEBAN	11	2016	GAMA	30.73	20.91	25.58	11.46	88.03	23.63	13.18	15.79	36.04	45.03
POR-INSEF	62	2019	AAMA	157.77	102.16	130.06	26.38	675.61	143.10	56.39	92.37	203.73	260.07
POR-INSEF	9	2020	AAMA	251.27	183.23	191.88	53.38	632.07	138.92	104.06	121.69	386.56	481.49
POR-INSEF	62	2019	GAMA	39.79	19.85	36.41	13.09	148.00	36.98	23.62	28.68	44.04	52.03
POR-INSEF	9	2020	GAMA	48.30	30.76	40.51	17.97	120.71	39.30	21.11	26.98	63.78	80.9
